# Tunable structural, optical, and electrical performance of PEMA/PMMA–CoCl₂ composites for advanced optoelectronics and energy storage applications

**DOI:** 10.1038/s41598-025-26091-0

**Published:** 2025-11-26

**Authors:** F. E. Hanash, Maha Aiiad Alenizi, G. M. Asnag, A. A. Al-Muntaser, M. O. Farea, Sadiq H. Khoreem, A. Y. Yassin

**Affiliations:** 1 Department of Mechatronics, College of Engineering and Information Technology, Emirates International University, 16881 Sana’a, Yemen; 2 Department of Physics, Faculty of Science, Sa’dah University, Sa’dah, Yemen; 3 Department of Physics, Faculty of Sciences-Arar, Northern Border University, P.O. Box 1321, 91431 Arar, Saudi Arabia; 4 Department of Mechatronics, College of Engineering and Smart Computing, Modern Specialized University, 12544 Sana’a, Yemen; 5 Center for Studies and Research, Amran University, 78428 Amran, Yemen; 6 Department of Physics, Faculty of Education and Applied Sciences at Arhab, Sana’a University, Sana’a, Yemen; 7 Department of Physics, Faculty of Science, Ibb University, Ibb, Yemen; 8 Department of Optometry and Visual Science, College of Medical Sciences, Al-Razi University, 71137 Sana’a, Yemen; 9 Department of Basic Sciences, Delta University for Science & Technology, Gamassa, Mansoura, Egypt

**Keywords:** PEMA/PMMA/CoCl_2_ composite films, Optical properties, Impedance analysis, Optoelectronic, Energy storage applications, Chemistry, Materials science, Nanoscience and technology

## Abstract

This work investigates the effects of CoCl₂ doping on the structural, optical, and impedance characteristics of PEMA/PMMA blends for the advancement of sophisticated polymeric multifunctional materials. Composite films containing CoCl₂ were prepared using the solution-casting method and characterized by various analytical techniques. XRD and FTIR analysis revealed reduced crystallinity with significant interactions in the doped samples, SEM revealed a homogeneous morphology with slight porosity as a result of filler incorporation. UV–Vis spectra have demonstrated a systematic decrease of both direct and indirect band gaps, which evidences an effective tuning of the electronic structure via controlled doping. Electrical studies have demonstrated a significant increase of ionic conductivity at higher CoCl₂ content, which was further supported by impedance spectra that have revealed a lower bulk resistance and better charge transport. Conducing equivalent-circuit models further confirmed and quantified these improvements in conductivity. This is a dual-polymeric (PEMA/PMMA) matrix doped with CoCl₂, hence attaining the simultaneous control of structural order, optical properties, and ionic transport each rarely observable in convention polymer films. The optimized 5.0 wt.% composite exhibits an excellent balance between the conductivity and structural stability and, hence, is considered a promising candidate for tunable optoelectronic and energy storage applications.

## Introduction

Due to increasing demand for more advanced materials that address more environmental needs, and emergent demands in technological development, human progress has been related and associated with the development of materials through the ages^1,2^. Polymers have become one of the key materials in modern technology owing to their extraordinary combination of physical, mechanical, and chemical properties^1,3^. Once crippled by a lack of scientific understanding of polymers, today they are being designed at the molecular level for advanced applications in virtually all fields of modern technology. Their multifunctional capabilities have been expanded greatly through the advancement of composites known as polymer matrix composites (PMCs) where polymers are combined with a number of various fillers or reinforcing agents into materials with higher strength, stability, and performance compared to the individual ingredients^1,3,4^. Science and technology are moving at an unprecedented pace, promoting intense searches for more and more new materials that would respond better to the growing demands put by immediate environments and functionality. Historically, materials development has shaped human progress—from the Stone and Bronze Ages through the present “Polymer Age”^1,2^. Polymer materials have become irreplaceable owing to their unique combination of properties: variable mechanical strength, flexibility, light weight, chemical stability, and low cost^1,3^. Once crippled by a lack of scientific understanding of polymers, today they are being engineered at the molecular level to deliver tailored performance for uses ranging from everyday consumer products to advanced aerospace and energy systems.

Further development of polymer matrix composites has advanced their properties. PMCs are materials in which polymers are combined with fillers or reinforcements to develop superior multifunctional behavior^1,3^. Recently, PMCs have increasingly substituted conventional materials such as metals/oxides and ceramics for a wide range of applications in transportation^3,4^, aerospace^5^, energy storage^6,8^, defense^7^, electronic devices^11,12^, and even food packaging^9,10^. This wide employment is accredited to their excellent strength-to-weight ratio, resistance against corrosion, flexibility in structural design, and superior thermal/dielectric stability of PMCs^13–15^. With the addition of either short or continuous fibers, fillers, or other reinforcement phases in a polymer matrix, engineers can make composites that boast of low weight, along with mechanical strength capable of sustaining extreme environments under operational conditions^15–17^.

Among the thermoplastic polymers, much interest was attracted by a thermoset-based system due to processability, recyclability, low moisture absorption, and relative low cost^17,18^. Blending different kinds of thermoplastic polymers is an effective method to combine the desirable properties of each component while avoiding the limitations. Such a combination of two structurally compatible polymers may be represented by a blend of PEMA and PMMA, which exhibit excellent miscibility^19–22^. PEMA provides flexibility and ionic conductivity, while PMMA can provide improvement in optical properties, stiffness, and thermal stability. By synthesizing their blends, one gets homogeneous films whose structural, optical, and electric properties can be tuned to a wide variety of advanced optoelectronic and electrochemical applications^22,23^.

In an effort to further improve functionality, metal salts have added to the polymer matrices for enhancing ionic conductivity, dielectric behavior, and efficiency of charge transportation^24–26^. Such addition of metal salt may suppress the crystallinity of polymers and enhance the amorphous phase, which results in easy migration of ions and improvement in electrochemical stability^27–29^. Among a variety of metal salts, there has been great interest in cobalt chloride (CoCl₂) because of its strong accepting ability, ability to form charge-transfer complexes, and tendency to interact with both amorphous and crystalline parts of the host matrix^30–32^. When incorporated into the polymer matrix, CoCl₂ coordinates with carbonyl groups, improving inter-chains connectivity, and establishing additional ionic-transport paths. Its uniform dispersion within the amorphous area promotes localized conduction channels; hence, the mobility of charges and dielectric performance improved^31,32^.

Although individual works have studied the influence of polymer blending or metals-salts doping separately, there remains a lack of comprehensive understanding of how these two modifications work synergistically to tune both the optical and electrical features of polymer-based films. Furthermore, conventional polymer thin films such as PEO suffer from high crystallinity and limited conductivity at ambient conditions, motivates the exploration of alternative polymer hosts^17,29^.

This study, in this context, will explore the synthesis of PEMA/PMMA–CoCl₂ composite films through a solution casting method and explore the effects of the addition of CoCl₂ on structural, optical, and electrical properties. The structural and property variations were analyzed by using techniques like XRD, FTIR, SEM, UV–Vis, and impedance spectroscopy. All these characterizations indicate a decrement trend in crystallinity with increased amorphous ratio and facilitated charge transport along with tunable optical properties as CoCl₂ concentration increases.

The originality of this work is found in the proof of a dual-polymer matrix coupled synergistically, PEMA/PMMA, with CoCl₂ filling toward the simultaneous control of structural order, optical band gap, and ionic transport, barely reported until now in the frame of conventional polymer electrolytes. Optimized 5.0 wt.% CoCl₂ composite exhibited the best balance among conductivity, transparency, and stability, establishing its potential to be used as a multifunctional material in next-generation optoelectronic and solid-state energy-storage applications.

## Experimental work

### Materials

PEMA (purity: 98.5%) with a molecular weight of 50 kg·mol⁻^1^ and a density of 1.119 g/cm^3^, and PMMA (purity: 99.5%) with a molecular weight of 120 kg·mol⁻^1^ and a density of 1.18 g/cm^3^, were purchased from Merck, Frankfurter Strasse 250, Darmstadt, Germany. Cobalt (II) chloride (CoCl₂, purity: 99%) was obtained from Hayashi Pure Chemical Co., Japan. Benzene (purity: 99.9%), used as the solvent for all samples, was obtained from El-Nasr Chemicals Co., Cairo, Egypt.

### Preparation of PEMA/PMMA–CoCl_2_ composite samples

PEMA/PMMA–CoCl₂ composite films were synthesized using solution casting method. Equal masses of PEMA and PMMA (50:50 wt.%) were dissolved in benzene under continuous magnetic stirring at ambient temperature for 9 h until a homogeneous solution was obtained.

Different amounts of CoCl₂ (0.0, 2.0, and 5.0 wt.%) were then incorporated to the polymer blend solutions and stirred for 3 h, followed by probe sonication (pulsed mode, 5 min) to ensure uniform dispersion of the salt. The prepared composite solutions were cast into clean glass Petri dishes and dried in an oven at 35 °C for two days to allow complete solvent evaporation. Following the drying process, the films were stored in a vacuum desiccator to prevent moisture absorption due to the hygroscopic nature of CoCl₂.

The measured film thicknesses were 59 ± 2 μm (pure blend), 66 ± 2 μm (2 wt.% CoCl₂), and 75 ± 2 μm (5 wt.% CoCl₂).

### Optimization of PEMA/PMMA blend matrix, solvent selection, and CoCl₂ concentrations

The 50:50 PEMA/PMMA ratios was strategically selected to provide optimal film-forming properties, solubility, mechanical stability, and environmental durability—essential requirements for optoelectronic and energy-storage applications. This composition exhibited excellent miscibility between PEMA and PMMA, producing smooth, flexible, and defect-free films consistent with literature reports^33–35^.

Benzene was chosen because it dissolves both PEMA and PMMA effectively, enabling uniform mixing and controlled film formation. Its moderate evaporation rate minimizes voids and cracks, yielding dense, stable films with improved interfacial contact between the polymer and CoCl₂ filler. While acetone and DMF can also dissolve the polymers, their different volatilities and polarities would alter the composite structure. Acetone’s rapid evaporation may cause surface roughness, whereas DMF’s slow drying and strong polarity could leave residual solvent and affect dielectric behavior. Benzene thus offered the best balance of solubility, volatility, and film uniformity.

To tailor the electrical and optical behavior of the PEMA/PMMA blend, CoCl₂ was incorporated at different concentrations. The 5.0 wt.% loading was identified as the optimal level for achieving enhanced functional performance and uniform filler dispersion. At lower concentrations (< 5.0 wt.%), the limited number of Co^2^⁺ coordination sites results in weak polymer–filler interactions and minimal improvement in charge transport or optical response. Conversely, higher concentrations (> 5.0 wt.%) promote particle agglomeration, increase viscosity, and disrupt polymer chain continuity, leading to phase separation and reduced film uniformity. Excess filler also introduces interfacial scattering and localized defect states that degrade optical transparency, dielectric stability, and mechanical integrity. Therefore, conductivity and optical properties improve only up to the 5.0 wt.% threshold, beyond which performance progressively deteriorates. This composition thus represents the best compromise between structural stability, ionic mobility, and processability**.** Notably, previous studies have also reported using a similar CoCl₂ concentration (5.0 wt.%) in polymeric composite systems, confirming its effectiveness in achieving optimal electro-optical properties^36–39^.

These optimized processing conditions ensured the formation of stable and homogeneous PEMA/PMMA–CoCl₂ films, making them suitable for multifunctional applications such as optoelectronic devices, solid-state capacitors, and battery separators.

### Characterization techniques

The structural, optical and electrical characterization of PEMA/PMMA–CoCl_2_ composite films was performed using various analytical techniques. X-ray diffraction (XRD) analysis was conducted on a PANalytical XPert PRO (Holland) diffractometer with Cu-Kα radiation (wavelength = 1.54 Å). The operating conditions for XRD analysis was 45 kV and 40 mA, over a 2θ range of 4°–50°. Mid-infrared spectra in the range of 4000 to 400 cm⁻^1^ were recorded using a Nicolet iS10 Fourier transform infrared (FTIR) spectrometer (USA). Optical absorption spectra were measured over the wavelength range 200–800 nm using a UNICAM UV–Vis spectrometer (England). The surface morphology and filler dispersion were examined with a JEOL JSM-6510LV scanning electron microscope (SEM) (USA) operated at an accelerating voltage of 20 kV. Frequency-dependent electrical and dielectric measurements were obtained using a 3532–50 LCR HiTESTER (Hioki, Japan) within the 0.1 Hz–20 MHz range.

Films thickness was measured using a digital micrometer, Model 7326, Mitutoyo (Tokyo, Japan), and each reported value represents the average of four measurements taken from different regions of the film to ensure precision and reproducibility.

## Results and discussions

### XRD scans

**Fig. 1:**
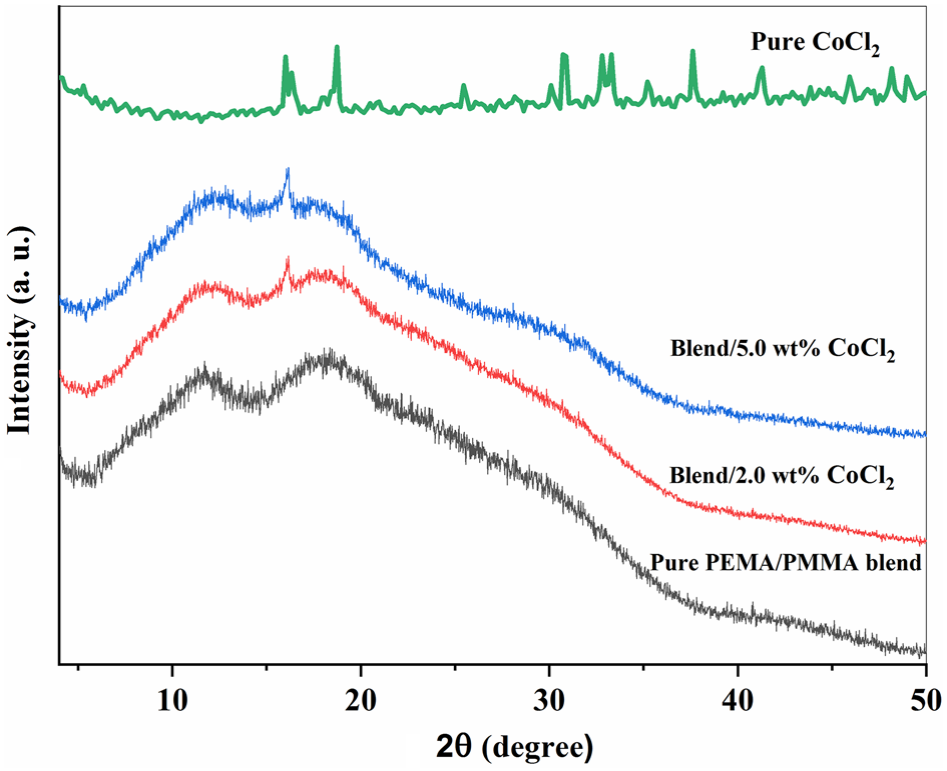
XRD patterns of pure CoCl₂ particles, the pure PEMA/PMMA blend, and its CoCl₂-doped composites containing 2.0 and 5.0 wt.% CoCl₂ filler.

The XRD patterns of pure CoCl₂ powder, the pure PEMA/PMMA blend, and the PEMA/PMMA–CoCl₂ composites with different filler concentrations are presented in Fig. 1. X-ray diffraction is a fundamental analytical technique in materials science, commonly employed to determine the crystallographic structure, unit-cell dimensions, and phase composition of solids^39^.

In the diffraction pattern of the pure CoCl₂ sample, there are a number of sharp and intense peaks, confirming its highly crystalline nature. The most prominent reflection appears at approximately 2θ = 18.7° and corresponds to the (012) plane of the rhombohedral CoCl₂ phase. This reflection gives the base orientation in the cobalt chloride lattice and therefore indicates that the atomic layers are well ordered in that particular direction. Additional characteristic reflections are observed at around 16.0°, 25.4°, 30.8°, 32.7°, 33.3°, 37.6°, 41.3°, 48.1°, and 48.9°, indexed to the (110), (104), (200), (211), (113), (220), (311), and (400) planes, respectively^40–42^. No extra peaks indicate that the CoCl₂ powder is phase-pure without detectable impurities^43^. These diffraction features are in good agreement with the standard data of crystalline CoCl₂ previously reported in the literature and thus confirm the correctness of phase identification. On the contrary, the XRD pattern of pure PEMA/PMMA blend exhibited two broad diffused halos centered around 11.8° and 18.4°, typical characteristics for amorphous polymers. Such broad peaks arise from a random distribution of the polymer chains and indicate the absence of long-range order. Diffused halo also reflects intermolecular interactions between PEMA and PMMA chains that influence molecular packing and confirms partial miscibility between the two polymers^44,45^. With the addition of CoCl₂, a new diffraction feature at around 16.0° and a number of weak secondary peaks of low intensity appear, confirming the uniform dispersion of the filler in the polymer matrix^44^. At higher CoCl₂ concentration, there is gradual broadening of the main halo and loss of general peak intensity due to its reduction in crystallinity and with higher amorphous character, indicating that CoCl₂ prevents the well ordering of the polymer chains in the lattice through introducing strain and microscopic defects. The main peak is followed by a broad amorphous halo showing higher disorderness and existence of amorphous–crystalline mixed region, which is a general characteristic of polymer–salt complex systems^38,44^.

Additionally, No significant shift in peak positions is observed in composite samples (curves 2–3), indicating that the addition of CoCl₂ does not change the essential amorphous nature of the blend. However, minor distortions of the diffraction lines suggest micro-defects or local strain due to coordination between Co^2^⁺ ions and the polymer functional groups. These distortions confirm strong polymer-filler interactions and partial destruction of the polymer network.

Overall, the XRD results demonstrate that cobalt chloride is well dispersed within the PEMA/PMMA matrix, effectively modifying its microstructure by reducing crystallinity and enhancing amorphous content. This structural transformation is beneficial for improving ion mobility and electrical conductivity, as the amorphous regions facilitate charge transport within the composite films^44,45^.

### FTIR spectroscopy spectra

**Fig. 2:**
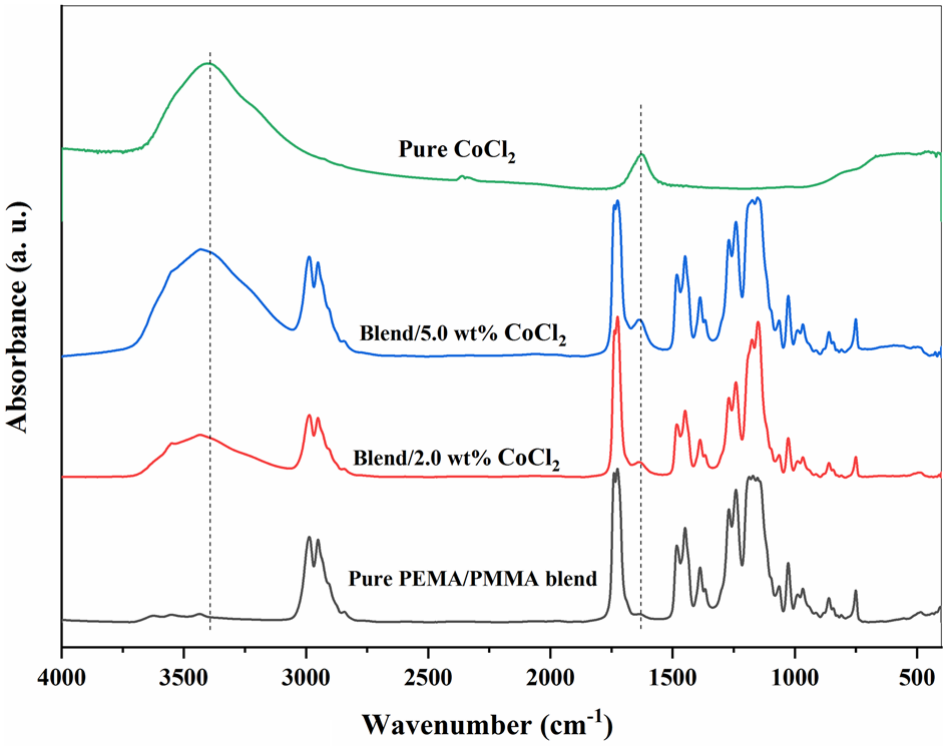
FTIR absorption spectra of pure CoCl₂, pure PEMA/PMMA blend, and its CoCl₂-doped composites containing 2.0 and 5.0 wt.% CoCl₂.

FTIR spectroscopy is an important analytical technique in studying polymer structures because it gives valuable information about the molecular interaction, bonding configurations, and possible complex formations between different polymer matrices or between a polymer blend and a dopant such as CoCl₂^46^. FTIR spectra of pure CoCl₂, pure PEMA/PMMA blend, and PEMA/PMMA–CoCl₂ composites are shown in Fig. 2. The spectrum of the pure PEMA/PMMA blend exhibits some characteristic absorption bands. The peaks at 2991 and 2951 cm⁻^1^ correspond to the symmetric and asymmetric stretching vibrations of the methylene (–CH₂–) groups of both polymers^47^. The prominent absorption band at 1725 cm⁻^1^ is due to the stretching of C=O of the ester groups of both PEMA and PMMA^48^. Both the polymers are consisted of ester functionality in their repeating unit so, this strong band is reflecting the combined contribution of the components^46,49^. The carbonyl (C=O) group also makes its contribution to the formation of polarons and bipolarons in the polymer matrix, which is important for charge transport in the composites^49^. Furthermore, additional peaks have been observed at 1487 and 1448 cm⁻^1^, attributed to C–H bending vibrations of the methyl (–CH₃) and methylene (–CH₂) groups from PMMA and PEMA, correspondingly^42^. The peak appearing at 1386 cm⁻^1^ is also ascribed to the C–H bending vibration, counting the absorption band at 1238 cm⁻^1^ for C–O–C stretching vibrations of the ester linkage. The band near 1170 cm⁻^1^ is attributed to the C–C stretching vibration of the PEMA backbone^46^. The general spectral profile of the blend confirms that PEMA and PMMA have good miscibility, as reflected in the merged and broadened absorption features reflecting interchain interactions between the two polymers^47^. With the addition of CoCl₂, significant changes are brought about in the composite samples’ spectra. Two new absorption bands have appeared at ca. 3404 cm⁻^1^ and 1634 cm⁻^1^, which could be assigned to O–H and C=C stretching^47,49^. The O–H band appearing at about 3404 cm⁻^1^ reveals some hydrogen bonding or moisture absorption, typical of such polymer-salt complexes, while the position appearing at 1634 cm⁻^1^ reflects the probable coordination of Co^2^⁺ ions with the carbonyl or unsaturated sites of the polymer backbone. This sort of coordination would change the local electron density around the C=O group with a view to improve polymer salt interaction. The addition of CoCl₂ also causes slight peak shifts and intensity variation, indicating that there are strong electrostatic interactions between Co^2^⁺ cations with polar groups –C=O and –C–O–C– of the polymer matrix. Such interactions cause disruption of the polymer packing, increase free volume, and subsequently increase chain flexibility, providing additional ionic transport pathways. Hence, the addition of CoCl₂ enhances ion mobility inside the blend, which, in turn, increases AC conductivity and dielectric performance^37,38^.

These results verify the creation of coordination complexes between PEMA/PMMA matrix and CoCl₂ filler, thus supporting previous literature evidence in the literature^47,49^. Moreover, increased interfacial interaction along with growing amorphous content is expected to facilitate improvement in charge transfer and ionic conduction, hence qualifying the composites for different optoelectronic and energy-storage applications at their solid state.

### UV/Vis. optical studies

**Fig. 3:**
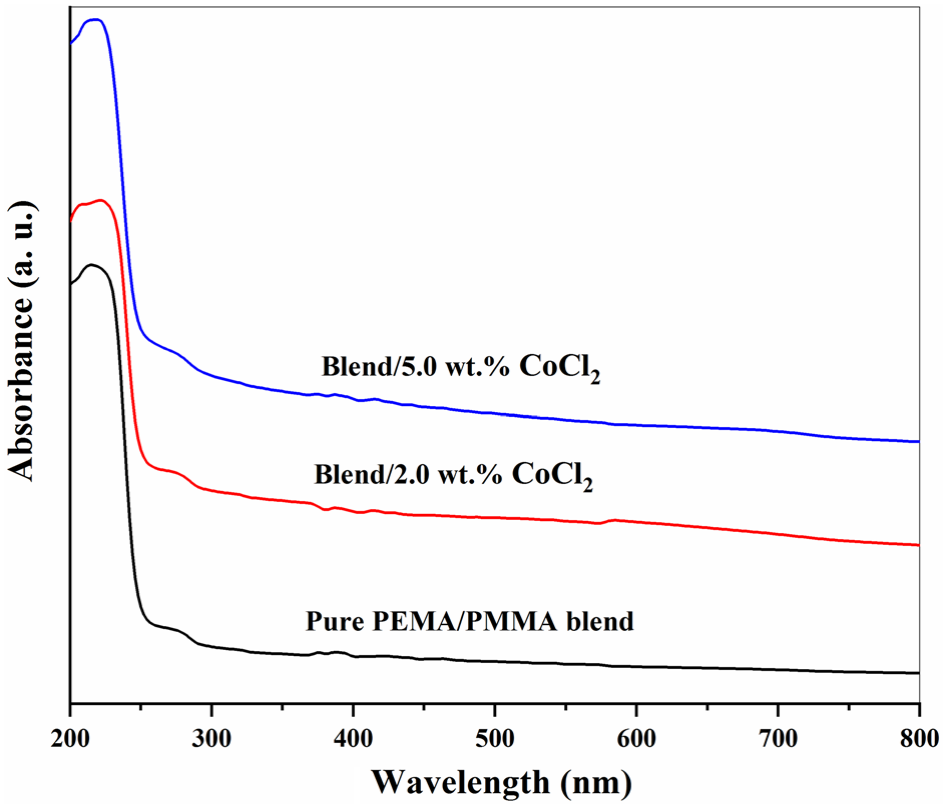
UV–Vis absorption spectra of pure PEMA/PMMA blend and its CoCl₂-doped composites containing 2.0 and 5.0 wt.% CoCl₂, recorded at room temperature.

Figure 3 UV–Vis absorption spectra of pure PEMA/PMMA blend and composite films containing different concentrations of CoCl₂. Pure PEMA/PMMA matrix exhibited a strong absorption peak near 212 nm, ascribed to the π → π* transition of the C=O group inside the polymer backbone, which is in good agreement with FTIR results^50,51^. This peak gradually shifts towards longer wavelengths (red shift) upon the addition of CoCl₂, indicating a decrease in the degree of crystallinity and forming new localized electronic states within the forbidden energy gap. These changes are associated with higher amorphous fraction, as evidenced by XRD study. Further, charge hopping via the higher amorphous fraction is enhanced and thus improves AC conductivity^26^. The coordination between Co^2^⁺ ions and polar functional groups of the PEMA/PMMA matrix, along with charge-transfer interactions, is plausible to be responsible for such behavior. Changing absorption intensity in composites with 2.0 wt.% and 5.0 wt.% CoCl₂ is connected with structural rearrangements in the polymer network that modify the optical energy band gap. Complexation of CoCl₂ with polymer chains increases structural defects and chain disorder, thus enhancing the segmental motion of the polymer backbone and narrowing the optical energy gap^51,52^.

The optical energy gap (E₉), a key factor that prescribes the optical and electronic behavior of materials, was determined using Tauc’s relation^51^.

1 $$(\alpha {\text{h}}\upsilon )^{{\text{n}}} = \, ({\text{h}}\upsilon - {\text{ E}}_{{\text{g}}} )$$

**Fig. 4:**
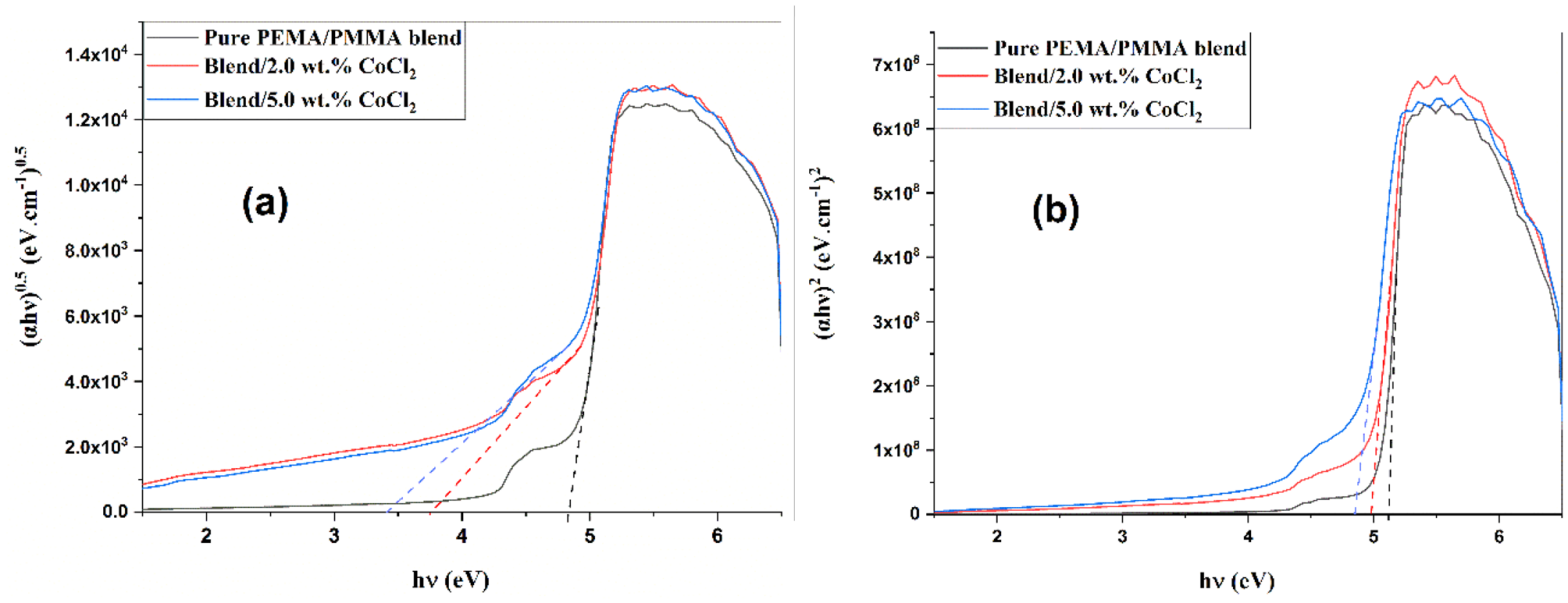
Tauc plots used to determine the optical energy gaps of PEMA/PMMA/CoCl_2_ composite samples: **a** Plot of (αhν)^0.5^ versus hν for the indirect energy gap and **b** Plot of (αhν)^2^ versus hν for the direct energy gap.

In this case, hυ is photon energy, α is the absorption coefficient, and B is a material-dependent constant; n = ½ or 2 for allowed indirect and direct transitions, respectively. The corresponding Tauc plots, (αhv)^1/2^ and (αhν)^2^ versus hν are shown in Fig. 4a and b.

The different estimated values of direct and indirect band-gap energies decrease from 5.12 eV and 4.83 eV for the pure PEMA/PMMA blend to 4.84 eV and 3.40 eV, respectively, for the composite containing 5.0 wt.% CoCl₂ as Table 1. The low value of E₉ in the doped films confirms increased disorder and a higher density of states in the band structure as a result of strong interactions between polymer and filler. As a result, these localized states further facilitate CTC between V.B. and C.B., leading to low-energy electronic transitions.

**Table 1:** Indirect and direct optical energy gaps (E_ind_ and E_di_) of PEMA/PMMA–CoCl₂ composite films at different CoCl₂ concentrations.

Concentrations of CoCl_2_ (wt.%)	E_ind_ (eV)	E_di_ (eV)
0.0	4.83	5.12
2.0	3.75	4.98
5.0	3.40	4.84

The coupling between the PEMA/PMMA chains and CoCl₂ particles modifies the electronic configuration through orbital hybridization and partial charge transfer, which alters the density of states and reduces the band gap. Additionally, possible confinement and quantum-size effects at the polymer–filler interface may further contribute to the observed shift. The reduced band gap enhances electronic polarization and charge-storage capability, thereby improving the dielectric and capacitive performance of the composites^17,26^.

The optical band-gap energies were determined from Tauc plots derived from UV–Vis absorption spectra. Both indirect and direct energy gaps decrease progressively with increasing CoCl₂ content from 4.83/5.12 eV for the pristine blend to 3.40/4.84 eV for the 5.0 wt.% composite indicating enhanced electronic interactions between CoCl₂ and the polymer matrix and confirming successful band-gap tuning through controlled doping.

### SEM analysis

**Fig. 5:**
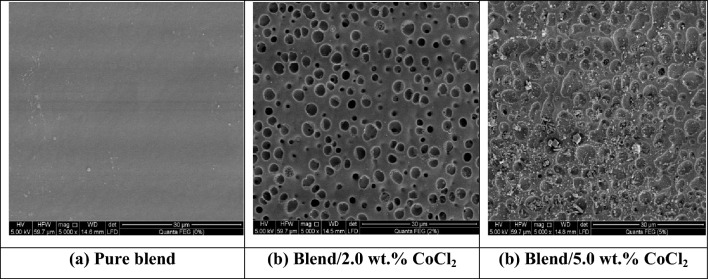
SEM micrographs of pure PEMA/PMMA blend and its CoCl₂-doped composites containing 2.0 and 5.0 wt.% CoCl₂, recorded at 5000× magnification.

Figure 5 presents the SEM micrographs of the PEMA/PMMA blend and its CoCl₂-doped composites at different CoCl_2_ contents. The micrograph of the pristine PEMA/PMMA matrix shows a smooth and homogeneous surface, indicating good miscibility between the two polymer phases. After the addition of CoCl₂, the surface morphology becomes noticeably rougher, with several craters and pores appearing across the film. These surface irregularities are most likely caused by the interaction between CoCl₂ particles and the polymer chains, which can locally disturb the homogeneous structure of the PEMA/PMMA blend and promote minor phase separation or localized chain degradation.

This pore formation can also be coupled to the hygroscopic nature of CoCl₂, which tends to absorb trace amounts of moisture in its preparation. During the drying stage, the rapid evaporation of solvent (benzene) followed by the removal of moisture may have left small voids or pores on the surface of the film^29,53^. To minimize these effects, all samples were oven-dried at 35 °C for 48 h and stored in a vacuum desiccator before SEM examination to prevent re-absorption of moisture. At the highest CoCl₂ concentration (5 wt.%), partial aggregation of the filler is evident, reflecting the limited miscibility and solubility of CoCl₂ within the polymer matrix at elevated loadings.

Such agglomeration is a common phenomenon in polymer–salt composites when the filler concentration exceeds the critical dispersion threshold, leading to localized stress points and minor surface heterogeneity. Strong ionic and dipole–dipole interactions between CoCl₂ molecules further promote clustering at higher contents^29,38,47^.

### AC conductivity (σ_ac_)

**Fig. 6:**
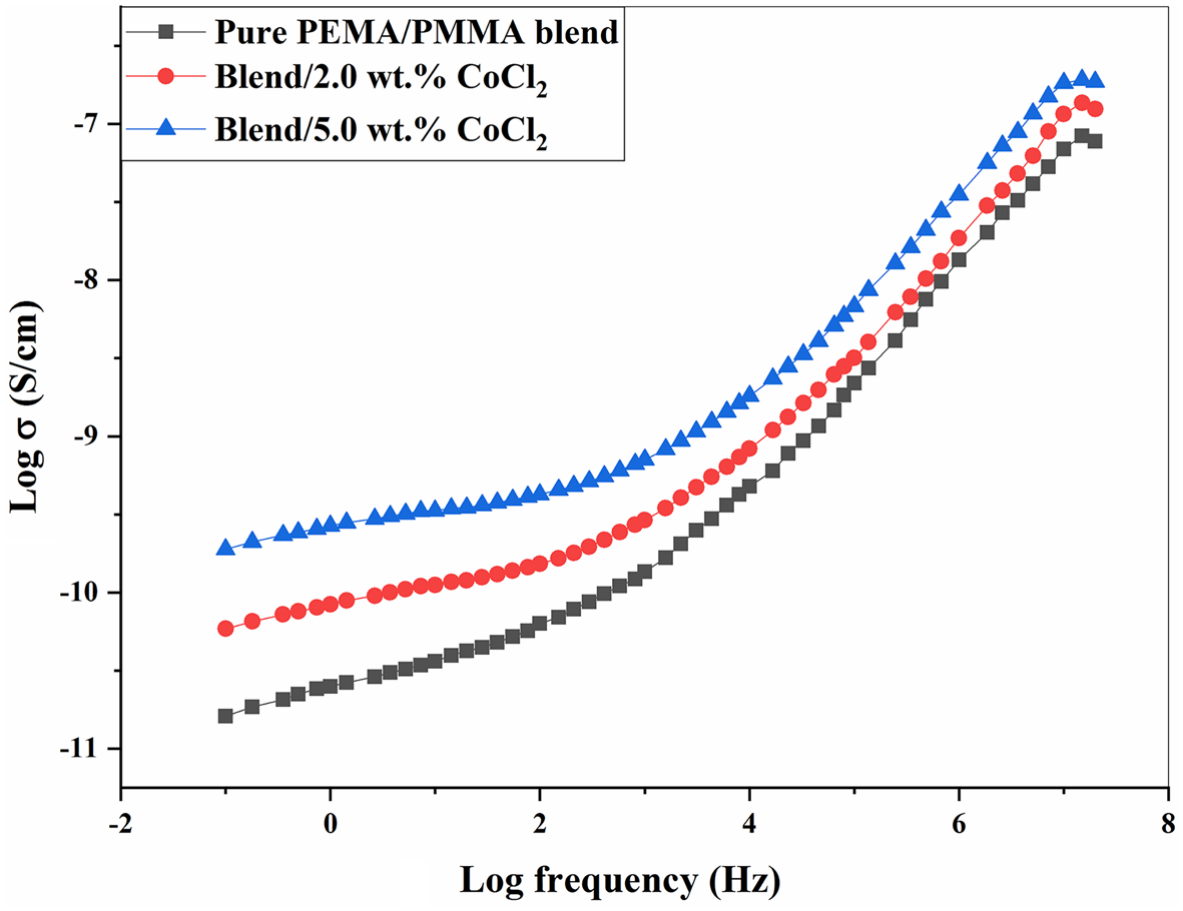
AC conductivity (σ_ac_) spectra of pure PEMA/PMMA blend and its CoCl₂-doped composites containing 2.0 and 5.0 wt.% CoCl₂ as a function of frequency at room temperature.

Figure 6 shows the AC conductivity of pure PEMA/PMMA blend filled with various concentration of CoCl_2_ at room temperature. The AC conductivity of PEMA/PMMA/CoCl_2_ composites at was calculated at room temperature using the following equation^54^:

2 $${\upsigma }_{ac}= \frac{L}{RA}$$

Where R represents bulk resistance (Ω), L is the sample thickness in centimeters (cm), and A denotes the electrode area in square centimeters (cm^2^). The AC conductivity in the plateau region at low frequencies indicates a DC-like conduction mechanism, while at high frequencies it appears in the dispersion region range, demonstrating frequency-dependent behavior. This transition can be explained based on the jump relaxation model, where free charge carriers at lower frequencies can move between adjacent sites via hopping. In the low-frequency region, the increased charge accumulation delays charge carrier movement, reducing electrical conductivity of PEMA/PMMA/CoCl_2_ composite films. As the frequency increases, the probability of successful hopping also increases, leading to the observed dispersion behavior, with the conductivity increasing attributed to the potential spatial charge of ions, or electrode polarization effect, which can be explained by bulk relaxation phenomena^33,41,54^. This behavior indicates a charge carrier hopping mechanism, where an alternating electric field moves and releases trapped carriers between localized states. The conductivity depends on frequency, increasing due to spatial charge or electrode polarization, indicating a charge carrier hopping mechanism. In addition, with increasing CoCl_2_ concentration, the value of σ_ac_ increases, as shown in Fig. 6. This increase is attributed to the higher numbers of mobile charge carriers introduced by the incorporated of CoCl_2_ into pure PEMA/PMMA blend. The addition of CoCl₂ leads to the dissociation of ionic species, contributing to a greater density of free ions, which, in turn, enhances the ionic conductivity of the composite, due to nature of interaction between the polymer chains and cobalt ion molecules as well as the load level of the dopant salt. Specifically, the Co^2^⁺ cations act as the dominant charge carriers, improving the overall conductivity of the system. This is due to the dramatic increase in mobile charge carriers that contribute to ion transport in the composites^42,47^. Previous study on CoCl₂-doped PVA polymer films showed that AC conductivity increases with CoCl₂ concentration, making these films suitable for electrochemical applications^55^. Similarly, research on PVA and HPMC blended with PVP and added CoCl₂ showed enhanced bulk conductivity with the addition of CoCl₂, which was attributed to the increased ionic species capacity induced by the additive^56,57^. In addition, the observed enhancement in AC conductivity after CoCl_2_ incorporation can be associated with changes in the structural and morphological of the polymer matrix**.** XRD analysis shows that CoCl_2_ disrupts the polymer chains order, reducing crystallinity area and increasing amorphous degree of PEMA/PMMA blend^26^. These structural modifications facilitate the transport of ions-charge carriers Co^2^⁺ between localized states via the reduction of energy barriers in charges’ mobility. The amorphous region provides larger free volume and higher segmental motion, enhancing the movement of Co^2^⁺ ions between localized states. Further supporting the disordered structure of polymer using XRD data in which the jump relaxation model predicts that the more available hopping sites progressively increase σₐc with frequency and the concentration of CoCl₂. All these frequency-dependent AC conductivity behaviors, together with structural changes induced by CoCl₂, support the ion transport mechanism in an amorphous matrix. The above-mentioned interplay between charge carrier mobility, polymer structure, and frequency response offers a broader understanding of the conduction mechanism in composites made of PEMA/PMMA/CoCl₂. This enhanced conductivity, believed to result from filler-induced changes in the polymer structure favorable for charge carrier transport, makes these composite samples attractive materials for future electronic applications^33,41^.

### Impedance spectroscopy

**Fig. 7:**
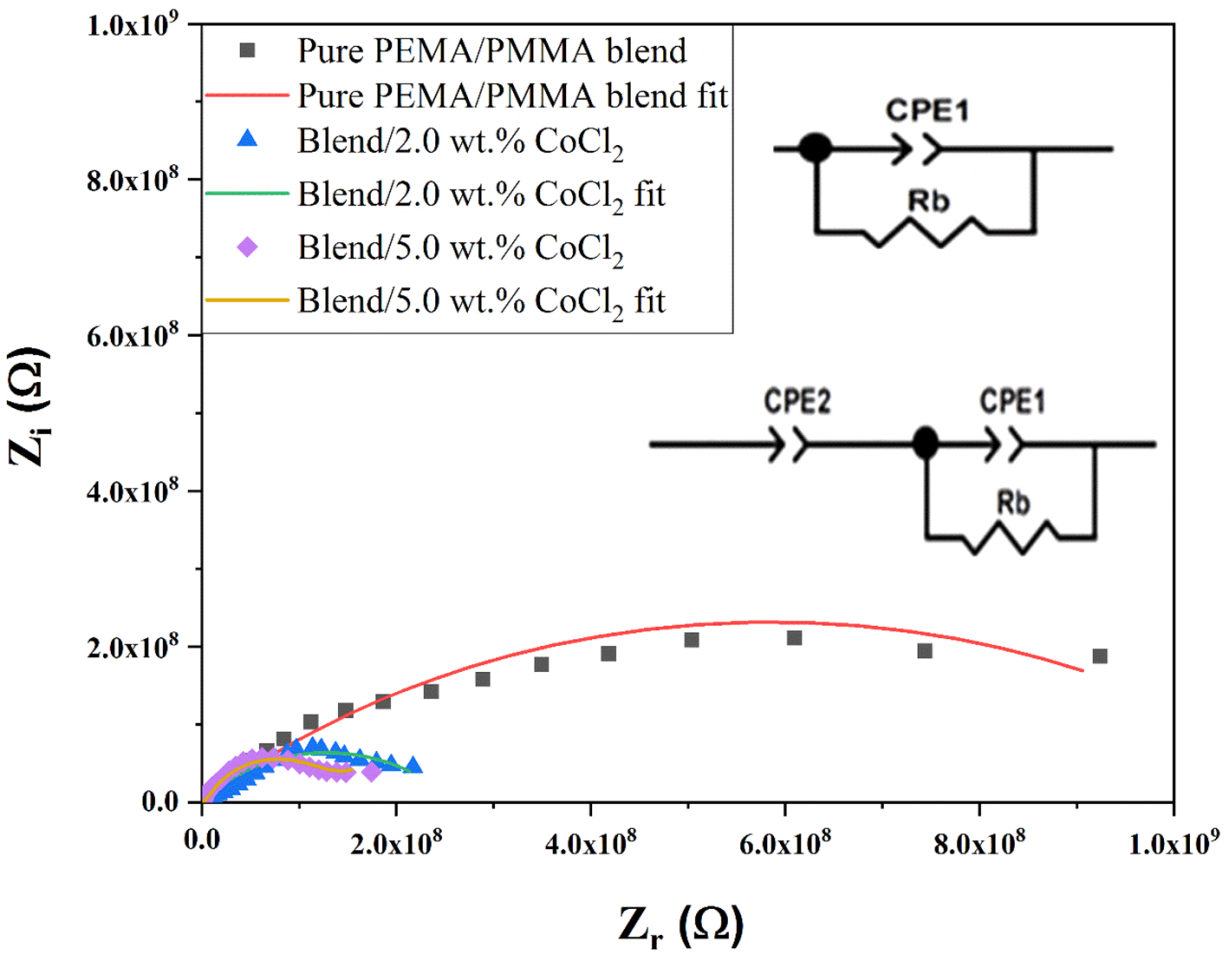
Nyquist plots and fitted equivalent circuit models for pure PEMA/PMMA blend and its CoCl₂-doped composites containing 2.0 and 5.0 wt.% CoCl₂ at room temperature. The corresponding equivalent circuit models illustrate the electrical behavior of the films, representing bulk resistance and interfacial capacitance elements.

To establish a correlation between the electrical properties and the microstructure, the impedance data were analyzed using equivalent-circuit modeling consisting of resistive and capacitive elements. Figure 7 presents the Nyquist plots (*Z*_r_ versus *Z*_*i*_) along with the corresponding equivalent-circuit parameters of the PEMA/PMMA–CoCl₂ composites at room temperature. The Nyquist plots exhibit a characteristic semicircle in the high-frequency region and an inclined line in the low-frequency region, typical of ion-conducting polymer systems. To interpret the impedance behavior, an equivalent electrical circuit (EEC) model was employed, comprising a bulk resistance (R_b_) and one or two constant-phase elements (CPE-1 or CPE-1 + CPE-2). The impedance of the constant-phase elements (CPE-1 and CPE-2) is defined by the following relation^58,59^:

3 $${Z}_{CPE}=\frac{1}{Q{\left(iw\right)}^{n}}$$

Where Q represents the numerical value of 1/Z at ω = 1 rad s⁻^1^, and n (0 ≤ n ≤ 1) denotes the phase factor, indicating the deviation from an ideal capacitor. The R_b_ values were obtained from the intersection points of the semicircle with the Z′-axis. The high-frequency semicircle corresponds to the bulk response of the composite, where R_b_ and CPE-1 describe the combined effects of the polymer matrix and the electrode–electrolyte interface^41,58,59^. With an increase in the concentration of CoCl₂, semicircle diameter decreases steadily and corresponds to a decrease in R_b_ and an increase in conductivity (σ). Such behavior arises from partial dissociation of CoCl₂ into Co^2^⁺ and Cl⁻ ions, increasing the population of mobile charge carriers in the polymer matrix. The coordination of Co^2^⁺ with the carbonyl group of PEMA and PMMA disrupts the packing of polymer chains and enhances segmental mobility due to an increase in the amorphous fraction of the polymer matrix and thereby enhances ion transport. Such structural alterations reduce the interfacial impedance and facilitate efficient charge conduction within the composite network.

CPE-2, representing interfacial polarization, dominates the low-frequency linear region. The double-layer capacitance that forms between the conducting electrodes and the blended electrolyte in both high- and low-frequency domains is linked to the CPE-2 response. In the equivalent circuit model, the constant phase element (CPE) is used instead of an ideal capacitor because the electrolyte exhibits pseudo-capacitive behavior^60^.

The EIS software fitted the impedance data; the equivalent-circuit model parameters in Table 2. Rb decreases from 1.17 × 10⁹ Ω (pure blend) to 9.96 × 10⁷ Ω for 5.0 wt.% CoCl₂, confirming enhanced charge transport due to increased ionic dissociation and percolation pathways^17,45^. The constant-phase element Q₁ rises from 3.23 × 10⁻^1^⁰ F up to 9.30 × 10⁻^1^⁰ F for greater charge storage and ion mobility. Correspondingly, the non-ideality factor, n₁, increases from 0.48 to 0.90, which is interpreted as a gradual transition from more resistive to predominantly capacitive behavior, consistent with increased polymer-salt interactions^26^. The appearance of CPE₂ at 5.0 wt% CoCl₂ with Q₂ = 1.62 × 10⁻⁸ F can be interpreted as a critical microstructural transition, proving the development of an interfacial polarization due to Co^2^⁺ ions aggregation at the critical CoCl₂ loading, space charge regions and ionic double layers formation. It also testifies to the beginning of diffusion-limited processes in the low frequency region^58,60,61^. The concomitant reduction of R_b_, rise of Q₁, and emergence of Q₂ signify a substantial improvement in ionic conductivity within the PEMA/PMMA–CoCl₂ system, showing the intense coupling linked between microstructure and electrical performance^17,51^.

**Table 2:** Equivalent-circuit fitting parameters obtained from impedance analysis of PEMA/PMMA–CoCl₂ composite films.

Polymeric samples	R_b_ (Ω)	Q_1_ (F)	n_1_	Q_2_ (F)	n_2_
Pure PEMA/PMMA blend	1.17 × 10^9^	3.23 × 10^–10^	0.48	–	–
Blend/2.0 wt.% CoCl_2_	2.57 × 10^8^	1.20 × 10^–9^	0.58	–	–
Blend/5.0 wt.% CoCl_2_	9.96 × 10^7^	9.30 × 10^–10^	0.90	1.62 × 10^–8^	0.35

Further validation of the impedance and conductivity results was made by benchmarking the electrical performance of the PEMA/PMMACoCl₂ composites against some representative polymer electrolyte systems, particularly the PEO-based material. Conventional PEO electrolytes typically exhibit room-temperature ionic conductivities in the range of 1.2 × 10⁻⁸ S·cm⁻^1^^62^, 6.9 × 10⁻^11^ S·cm⁻^1^^33^, 3.88 × 10⁻^11^ S·cm⁻^1^^63^ and 3.41 × 10⁻^11^^64^, values that are largely limited by the high crystallinity and restricted segmental motion of polymer chains at ambient conditions^33^.

In contrast, the PEMA/PMMA–CoCl₂ composites developed in this study exhibit conductivities on the order of 10⁻⁷ S·cm⁻^1^ at the lowest frequency and 10⁻^1^⁰ S·cm⁻^1^ at 10 MHz, accompanied by a markedly lower bulk resistance (R_b_). Similar improvements in ionic conductivity upon CoCl₂ incorporation have been reported for other polymeric systems, where conductivities increased to the order of 10⁻⁸ S·cm⁻^1^ after CoCl₂ addition^36,38^. These results confirm that introducing CoCl₂ enhances ion mobility and polymer-segmental motion by increasing the amorphous content of the host matrix and facilitating more efficient charge transport.

Various factors may be synergistically combined and contribute to its better performance against the present system:

(i) PEMA/PMMA heterogeneous blend successfully eliminates crystallinity, which enhances the ionic conductivity effectively.
(ii) Inclusion of CoCl₂ introduces Lewis-acidic centers which promote salt dissociation and increase the number of mobile Co^2^⁺ and Cl⁻ ions.
(iii) Strong polymer-filler interactions formed efficient ion-transport pathways with minimal interfacial resistance.

With regard to conventional PEO-based electrolytes, the PEMA/PMMA–CoCl₂ system exhibited higher ionic conductivity and lower activation energy, as well as superior electrochemical stability, which thus demonstrates its potential in solid-state capacitors and flexible energy-storage devices, and optoelectronic applications. This benchmarking discussion was added into the revised manuscript to point out relative advantages and technological significance of the developed composite films.

The parameters were extracted by fitting Nyquist plots to an equivalent-circuit model consisting of bulk resistance (R_b_) and constant-phase elements (Q₁, n₁, Q₂, n₂). The reduction in R_b_ and increase in Q₁&Q_2_ with CoCl₂ addition demonstrate improved ionic conductivity and interfacial polarization within the polymer matrix.

## Conclusion

New solid polymer composite electrolytes based on a PEMA/PMMA blend doped with cobalt chloride (CoCl₂) were successfully prepared using the solution-casting method. FTIR and XRD analyses confirmed effective salt–polymer complexation and the strong solvating ability of PEMA/PMMA blend. UV–Vis spectroscopy showed red shift and intensity change due to reduced crystallinity, modified electronic states, and a narrower optical band gap. The SEM observations proved to have a smooth surface morphology and a homogeneous dispersion of CoCl₂ within the polymer matrix, which indicated good interfacial compatibility. The AC conductivity increased with both frequency and CoCl₂ concentration in electrical studies, due to the formation of an interconnected ion-transport pathway inside the polymer matrix. This further got confirmation from the impedance studies through Nyquist plots by showing a remarkable decrease in R_b_ from 1.17 × 10⁹ Ω for the pure blend to 9.96 × 10⁷ Ω for the composite containing 5.0 wt.% CoCl₂. These studies confirm that the incorporation of CoCl₂ effectively modifies the structural, optical, and electrical properties of the PEMA/PMMA matrix. The optimized composite demonstrates a well-balanced combination of reduced optical band gap, enhanced ionic conductivity, and improved dielectric response. Such tunable physicochemical performance highlights the potential of PEMA/PMMA–CoCl₂ composites for advanced energy-storage and optoelectronic applications.

## Data Availability

The original data presented in the study are included in the article; further inquiries can be directed to the corresponding author.

## References

[1] Oladele, I. O., Omotosho, T. F. & Adediran, A. A. Polymer-based composites: an indispensable material for present and future applications. *Int. J. Polym. Sci.* **2020**, 8834518 (2020).

[2] Oladele, I. O., Omotosho, T. F., Ogunwande, G. S. & Owa, F. A. A review on the philosophies for the advancement of polymer-based composites: past, present and future perspective. *Appl. Sci. Eng. Prog.* **14**, 553–579 (2021).

[3] Chaudhary, V. & Ahmad, F. A review on plant fiber reinforced thermoset polymers for structural and frictional composites. *Polym. Test.* **91**, 106792 (2020).

[4] Cherusseri, J. et al. *Polymer-Based Composite Materials: Characterizations* 37–77 (Springer, Berlin Heidelberg, 2016).

[5] Mishra, R. K., Chianella, I., Sarkar, J., Nezhad, H. Y. & Goel, S. Nanostructured ZnO-CQD hybrid heterostructure nanocomposites: synergistic engineering for sustainable design, functional properties, and high-performance applications. *ChemNanoMat.* **10**, e202400020 (2024).

[6] Costa, C. M., Lee, Y. H., Kim, J. H., Lee, S. Y. & Lanceros, S. L. Recent advances on separator membranes for lithium-ion battery applications: From porous membranes to solid electrolytes. *Energy Stor. Mater.* **22**, 346–375 (2019).

[7] Siengchin, S. A review on lightweight materials for defence applications: Present and future developments. *Def. Tech.* **24**, 1–17 (2023).

[8] Hema, S., Chandran, G. U., Sajith, M., Sulthan, K. R. & Sambhudevan, S. Polymer Blend Nanocomposites with CNTs for Energy Storage Applications. In *Polymer Blend Nanocomposites for Energy Storage Applications* 241–270 (Elsevier, 2023).

[9] Attaran, S. A., Hassan, A. & Wahit, M. U. Materials for food packaging applications based on bio-based polymer nanocomposites. *J. Thermoplast. Compos. Mater.* **30**, 143–173 (2015).

[10] Tarabiah, A. E. et al. Enhanced structural, optical, electrical properties and antibacterial activity of PEO/CMC doped ZnO nanorods for energy storage and food packaging applications. *J. Polym. Res.* **29**, 167 (2022).

[11] Chang, C. et al. A narrow-bandgap donor polymer for highly efficient as-cast non-fullerene polymer solar cells with a high open circuit voltage. *Org. Electron.* **58**, 82–87 (2018).

[12] Saeed, A. et al. Enhanced the structural, optical, electrical, and dielectric properties of PEO/CMC blend via TiO2 and ZnO nanoceramics: nanocomposites for capacitor applications. *J. Sol-Gel Sci. Technol.* **115**, 732–751 (2025).

[13] Chohan, J. S., Boparai, K. S., Singh, R. & Hashmi, M. S. J. Manufacturing techniques and applications of polymer matrix composites: A brief review. *Adv. Mater. Process. Technol.* **8**(1), 884–894 (2022).

[14] Begum, S., Fawzia, S. & Hashmi, M. S. J. Polymer matrix composite with natural and synthetic fibres. *Adv. Mater. Process. Technol.* **6**, 547–564 (2020).

[15] Mahesh, V., Joladarashi, S. & Kulkarni, S. M. A comprehensive review on material selection for polymer matrix composites subjected to impact load. *Def. Tech.* **17**, 257–277 (2021).

[16] Oladele, I. O., Akinwekomi, A. D., Ibrahim, I. O., Adegun, M. H. & Talabi, S. I. Assessment of impact energy, wear behavior, thermal resistance and water absorption properties of hybrid bagasse fiber/CaCO_3_ reinforced polypropylene composites. *Int. Polym. Process.* **36**, 205–212 (2021).

[17] Morsi, M. A. et al. Reinforced PEO/Cs polymers blend with Al2O3/TiO2 hybrid nanofillers: Nanocomposites for optoelectronics and energy storage. *J. Energy Storage.* **88**, 111554 (2024).

[18] Kumar, V. V. et al. A review of recent advances in nanoengineered polymer composites. *Polym.* **11**, 644 (2019). 10.3390/polym11040644 PMC6523580 30970621

[19] Mani, S., Patwardhan, S., Hadkar, S., Mishra, K. & Sarawade, P. Effect of polymer concentration on optical and electrical properties of liquid crystals for photonic applications. *Mater. Today: Proc.* **62**, 7035–7039 (2022).

[20] Malik, P., Chauhan, G., Kumar, P. & Deep, A. Effect of polymer concentration on the electro-optical, dielectric and photoluminescence properties of confined ferroelectric liquid crystals composites. *Liq. Cryst.* **49**, 2008–2018 (2022).

[21] Wani, A. A. et al. Critical review on composite-based polymer electrolyte membranes toward fuel cell applications: Progress and perspectives. *Energy Fuels.* **38**, 18169–18193 (2024).

[22] Ramesh, S., Uma, O., Shanti, R., Yi, L. J. & Ramesh, K. Preparation and characterization of poly (ethyl methacrylate) based polymer electrolytes doped with 1-butyl-3-methylimidazolium trifluoromethanesulfonate. *Meas.* **48**, 263–273 (2013).

[23] Reinoso, D. M. & Frechero, M. A. Strategies for rational design of polymer-based solid electrolytes for advanced lithium energy storage applications. *Energy Storage Mater.* **52**, 430–464 (2022).

[24] Tawansi, A., Oraby, A. H., Abdelrazek, E. M., Ayad, M. I. & Abdelaziz, M. Effect of local structure of MnCl2-filled PVDF films on their optical, electrical, electron spin resonance, and magnetic properties. *J. Appl. Polym. Sci.* **70**, 1437–1445 (1998).

[25] Abdelrazek, E. M. & Ragab, H. M. Spectroscopic and dielectric study of iodine chloride doped PVA/PVP blend. *Indian J. Phys.* **89**, 577–585 (2015).

[26] Saeed, A. et al. Structural, optical, and electrical characteristics of HPMC/PVA-I2O5 composites: Fabrication and performance analysis for energy storage applications. *J. Energy Storage.* **96**, 112765 (2024).

[27] Rajendran, S., Prabhu, M. R. & Rani, M. U. Characterization of PVC/PEMA based polymer blend electrolytes. *Int. J. Electrochem. Sci.* **3**, 282–290 (2008).

[28] Mekky, A. B. H. Investigation of the influence of Br- and As-doped silica single-wall nanotubes: Hartree-Fock method. *Bull. Mater. Sci.* **41**, 164 (2018).

[29] Abdelghany, A. M., Oraby, A. H. & Asnag, G. M. Structural, thermal and electrical studies of polyethylene oxide/starch blend containing green synthesized gold nanoparticles. *J. Mol. Struct.* **1180**, 15–25 (2019).

[30] Wadatkar, N. S. & Waghuley, S. A. Studies on properties of as-synthesized conducting polythiophene through aqueous chemical route. *J. Mater. Sci. Mater Electron.* **27**, 10573–10581 (2016).

[31] Alghunaim, N. S. Spectroscopic analysis of PMMA/PVC blends containing CoCl_2_. *Results Phys.* **5**, 331–336 (2015).

[32] Abdelhameed, D., Morsi, M. A. & Elsisi, M. E. Impact of CoCl_2_ on the structural, morphological, optical, and magnetic properties of PCL/PVC blend for advanced spintronic/optoelectronic applications. *Ceram. Int.* **51**, 18713–18722 (2025).

[33] Rajeh, A., Ragab, H. M. & Abutalib, M. M. Co doped ZnO reinforced PEMA/PMMA composite: Structural, thermal, dielectric and electrical properties for electrochemical applications. *J. Mol. Struct.* **1217**, 128447 (2020).

[34] Abdelrazek, E. M. et al. Structural, optical, thermal, morphological and electrical studies of PEMA/PMMA blend filled with CoCl_2_ and LiBr As mixed filler. *J. Electron. Mater.* **49**, 6107–6122 (2020).

[35] Rawal, S. Poly (Ethyl Methacrylate) (PEMA) and its composites for various applications: Background, recent progress and future challenges. *Macromol. Symp.* **413**, 2300096 (2024).

[36] Chapi, S. Influence of Co^2+^ on the structure, conductivity, and electrochemical stability of poly(ethylene oxide)-based solid polymer electrolytes: Energy storage devices. *J. Electron. Mater.* **50**, 1558–1571 (2021).

[37] Chapi, S. & Devendrappa, H. Influence of cobalt (II) chloride catalysed on the thermal and optical characterization of PEO based solid polymer electrolytes. *J. Res. Updates Polym. Sci.* **3**, 205 (2014).

[38] Ahmeda, R. M., Ibrahiem, A. A. & El-Said, E. A. Effect of cobalt chloride as filler and PVP on the optical properties of PVA/PEG/PVP blends. *Opt. Spectrosc.* **128**, 642–655 (2020).

[39] Rameshkumar, C., Sarojini, S., Naresh, K. & Subalakshmi, R. Preparation and characterization of pristine PMMA and PVDF thin film using solution casting process for optoelectronic devices. *J. Surf. Sci. Technol.* **33**, 12–18 (2017).

[40] Al-Hakimi, A. N. et al. Enhancing the structural, optical, thermal, and electrical properties of PVA filled with mixed nanoparticles (TiO_2_/Cu). *Crystals* **13**, 135 (2023).

[41] Atta, M. R., Alsulami, Q. M., Asnag, G. M. & Rajeh, A. Enhanced optical, morphological, dielectric, and conductivity properties of gold nanoparticles doped with PVA/CMC blend as an application in organoelectronic devices. *J. Mater. Sci.: Mater Electron.* **32**, 10443–10457 (2021).

[42] El Gohary, H. G. et al. Modification and development of optical, thermal, dielectric properties and antibacterial activity of PVA/SA blend by Ag/Se nanofillers: nanocomposites for energy storage devices and food packaging applications. *Polym. Test.* **129**, 108258 (2023).

[43] Kaushik, A. et al. Influence of Co doping on the structural, optical and magnetic properties of ZnO nanocrystals. *J. Alloys Compd.* **578**, 328–335 (2013).

[44] Abdelrazek, E. M. Influence of FeCl_3_ filler on the structure and physical properties of polyethyl-methacrylate films. *Phys. B: Condens. Matter.* **400**, 26–32 (2007).

[45] Millan, W. M., Toledanothompson, T. T., Arriaga, L. G. & Smit, M. A. Characterization of composite materials of electroconductive polymer and cobalt as electrocatalysts for the oxygen reduction reaction. *Int. J. Hydrogen Energy* **34**, 694–702 (2008).

[46] Abdelrazek, E. M. & Elashmawi, I. S. Characterization and physical properties of CoCl_2_ filled polyethyl-methacrylate films. *Polym. Compos.* **29**, 1036–1043 (2008).

[47] El Gohary, H. G., Qahtan, T. F., Alharbi, H. G., Asnag, G. M. & Waly, A. L. Studies of the structural, optical, thermal, electrical and dielectric properties of a polyvinyl alcohol/sodium alginate blend doped with Cu nanoparticles and ZnO nanorods as hybrid nanofillers for use in energy storage devices. *J. Environ. Polym. Degrad.* **31**, 2930–2940 (2023).

[48] Wadatkar, N. S. & Waghuley, S. A. A novel studies on electrical behaviour of chemically synthesized conducting polyindole. *Indian J. Phys.* **92**, 1551–1559 (2018).

[49] Saeed, A. et al. Enhancing optical, structural, thermal, electrical properties, and antibacterial activity in chitosan/polyvinyl alcohol blend with ZnO nanorods: polymer nanocomposites for optoelectronics and food/medical packaging applications. *Polym. Bull.* **81**, 11645–11670 (2024).

[50] Saeed, A. et al. A comprehensive study on structural, optical, electrical, and dielectric properties of PVA-PVP/Ag-TiO2 nanocomposites for dielectric capacitor applications. *J. Alloys Compd.* **977**, 173412 (2024).

[51] Saron, K. M. A. et al. Controlling the dielectric and optical properties of polyvinyl alcohol/polyethylene glycol blends by adding copper oxide nanoparticles for application in energy storage devices. *J. Sol-Gel Sci. Technol.* **109**, 757–772 (2024).

[52] Askary, A. E. et al. Synthesis of nanostructured Bi_2_O_3_NPs using laser ablation technique and its effect on the optical, thermal, and conductivity characterization of the PEO/CMC blend. *J. Polym. Res.* **29**, 193 (2022).

[53] Reddy, M. R. et al. X-RD, SEM, FT-IR, DSC Studies of polymer blend films of PMMA and PEO. *Mater. Today Proc.* **3**, 3713–3718 (2016).

[54] Al-Muntaser, A. A. et al. Tuning the structural, optical, electrical, and dielectric properties of PVA/PVP/CMC ternary polymer blend using zno nanoparticles for nanodielectric and optoelectronic devices. *Opt. Mater.* **140**, 113901 (2023).

[55] Anyaogu, I. D., Nwanya, A. C., Ezema, F. I. & Ejikeme, P. M. Cobalt ion-doped polyvinyl alcohol: A promising multivalent polymer electrolyte system for aluminium—Air battery application. *Hybrid Adv.* **8**, 100369 (2025).

[56] Baraker, B. M. & Lobo, B. AC and DC electrical studies on cobalt chloride doped PVA–PVP blend films. *AIP Conf. Proc.* **2244**, 080015 (2020).

[57] Abdallah, E. M. et al. α-MoO_3_ nanobelts boosted the structural, optical, thermal, and dielectric properties of PEO/PVP blends for emerging optoelectronic/energy-storage applications. *ACS Omega* **1512**, 36396–36411 (2025). 10.1021/acsomega.5c04734 PMC12368632 40852228

[58] Saeed, A. et al. Influence of zinc acetate on HPMC/CMC polymer blend: Investigation of their composites’ structural, optical, and dielectric properties for dielectric capacitor applications. *Inorg. Chem. Commun.* **171**, 113536 (2025).

[59] Sunitha, V. R. & Radhakrishnan, S. Impedance and dielectric studies of nanocomposite polymer electrolyte systems using MMT and ferroelectric fillers. *Ionics* **22**, 2437–2446 (2016).

[60] Alenizi, M. A. et al. Structural, optical, electrical, and dielectric properties of HPMC/PVP blend reinforced with I_2_O_5_ for optoelectronics and energy storage applications. *J. Polym. Res.* **31**, 332 (2024).

[61] Khalil, R. Impedance and modulus spectroscopy of poly(vinyl alcohol)-Mg[ClO_4_]_2_ salt hybrid films. *Appl. Phys. A* **123**, 422 (2017).

[62] Ibrahim, P. A. et al. Glycerol as a multifunctional modifier: Synergistic enhancement of electrical and structural properties in PEO:NaF electrolytes. *J. Electron. Mater.* **54**, 5478–5492 (2025).

[63] Sengwa, R. J. & Choudhary, S. Dielectric and electrical properties of PEO–Al_2_O_3_ nanocomposites. *J. Alloys Compd.* **701**, 652–659 (2017).

[64] Morsi, M., Rajeh, A. A. & Al-Muntaser, A. A. Reinforcement of the optical, thermal and electrical properties of PEO based on MWCNTs/Au hybrid fillers: nanodielectric materials for organoelectronic devices. *Compos. B Eng.* **173**, 106957 (2019).

